# Application of targeted high-throughput sequencing as a diagnostic tool for neonatal genetic metabolic diseases following tandem mass spectrometry screening

**DOI:** 10.3389/fpubh.2024.1461141

**Published:** 2024-12-24

**Authors:** Guihua Lai, Qiying Gu, Zhiyong Lai, Haijun Chen, Xiangwen Tu, Junkun Chen, Jungao Huang

**Affiliations:** Central Laboratory, Ganzhou Maternal and Child Health Hospital, Ganzhou, Jiangxi, China

**Keywords:** genetic diagnosis, inborn errors of metabolism, newborn screening, targeted sequencing, tandem mass spectrometry

## Abstract

**Background:**

Tandem mass spectrometry (MS/MS) is a crucial technique for detecting inborn errors of metabolism (IEM) in newborns. However, the high false positive rate poses challenges in diagnosing specific types of diseases. Therefore, this study aimed to evaluate the role of targeted next-generation sequencing (NGS) in the accurate diagnosis of positive samples identified through MS/MS screening.

**Methods:**

A cohort study of 260,915 newborns was conducted from January 2018 to June 2023 in Ganzhou City, southern China. Heel blood samples were collected within 72 h of birth and subjected to MS/MS analysis. Infants with positive MS/MS results underwent targeted NGS to confirm the diagnosis and identify genetic variants.

**Results:**

Among 1,265 suspected cases with positive MS/MS results, 73 were confirmed by NGS, and 12 were identified as carriers of recessive diseases. The overall incidence rate was 1 in 3,574, effectively ruling out 94.2% (1,192/1,265) of the MS/MS false-positive. We found 76 variants in 18 genes associated with 15 types of IEM. Among these, 64.47% (49/76) were pathogenic, 10.53% (8/76) were likely pathogenic. Remarkably, 7.89% (6/76) were identified as novel variants. Variants in *SLC22A5* (NM_003060.4) gene was most prevalent, accounting for 41% (77/188), with hotspot variants including c.51C > G, c.1400C > G, and c.338G > A.

**Conclusion:**

Targeted NGS technology can serve as a crucial diagnostic tool for neonatal genetic metabolic diseases following MS/MS screening. Additionally, we identified IEM variant hotspots and some novel variants in our region, which are the underlying causes of disease in patients with IEM.

## 1 Introduction

Newborn screening (NBS) is a successful public health project that employs advanced testing techniques to detect some serious inherited metabolic diseases in newborns. This allows for early diagnosis and treatment before clinical manifestations occur, thereby preventing irreversible damage in children. According to statistics, genetic diseases occur in 3–5% of live births (1). Since Guthrie and Susi (2) first reported the bacterial inhibition test for phenylketonuria (PKU) screening in 1963, NBS has gained global recognition and is now a crucial tool in reducing neonatal morbidity and mortality. The implementation of NBS not only provides immediate health benefits for children diagnosed and treated early but also enables their participation in social activities and alleviates the burden on families. Traditional biochemical screening is currently the mainstream NBS method, including tandem mass spectrometry (MS/MS), electrophoresis technology, enzymology, immunology, and electrophoresis technology-high-pressure liquid chromatography (3). MS/MS technology is characterized by its high efficiency, sensitivity, and convenience, enabling early disease diagnosis (4). However, the spectrum of diseases tested by blood MS/MS is limited, and different diseases can result in elevations of the same metabolites, blood MS/MS testing has limited usefulness in accurate disease diagnosis. Moreover, metabolites can be influenced by various factors such as diet, underlying diseases, and preterm birth, potentially leading to false-positive and false-negative results, requiring further diagnosis (5).

In recent years, with the development of DNA sequencing technology, the focus on inborn errors of metabolism (IEM) screening technology has shifted from the metabolite level to the genetic level. Next-generation sequencing (NGS) technology was employed to discover the genetic factors of thousands of genetic diseases. Therefore, NGS is valuable for genotyping and detecting the genetic factors of IEM. By comprehensively assessing IEM based on the quantification of metabolites and genetic variants, NGS can effectively improve the accuracy of IEM screening, compensating for the limitations of MS technology (6).

In this study, we analyzed data from the NBS program with MS/MS over the past 6 years. Target NGS of genes in a custom panel was employed as a second critical step to diagnose high-risk infants identified by MS/MS, aiming to provide a definitive genetic diagnosis and determine IEM’s genetic characterization. This work has enhanced the quality of NBS programs, providing a more accurate diagnosis for children with IEM and consequently enabling more precise targeted therapy.

## 2 Materials and methods

### 2.1 Study design and participants

From January 2018 to June 2023, a total of 260,915 newborns underwent screening for IEM at the Ganzhou Maternal and Child Health Hospital in Jiangxi Province, China. Among these, 1,265 infants tested positive and received genetic diagnoses through NGS. Subjects were all newborns who had completed 72 h after birth and had been fed adequately at least eight times. Other inclusion criteria were complete medical history. Additionally, newborns were excluded if they were undergoing emergency surgery or external blood transfusion. The clinical characteristics of newborns with suspected IEMs were all fully understood by a single physician. The clinical data included sex, major clinical features, and outcomes of IEM. The confirmatory tests vary depending on the disease, including genetic testing or blood biochemical indices testing, enzyme activities testing and urine organic acids analysis, etc. Pretest counseling was performed by physicians. The study was approved by the ethics committee of the Ganzhou Maternal and Child Health Hospital (2020001). The legal guardians of the participating infants gave their written informed consent for their children to be included in the study.

### 2.2 MS/MS screening method

Heel blood samples were collected from newborns, dripped on filter paper (Schleicher & Schue11 903, Wallac OY Turku, Finland), and dried naturally at room temperature. Dried blood spots were pretreated using a non-derivative MS/MS kit per the manufacturer’s instructions (Fenghua, China) and then analyzed using a MS/MS system (Acquity UPLC-TQD, MA). Newborns with abnormal amino acid or carnitine (free carnitine and acylcarnitine) indices were recalled for recollection of heel blood (filter paper dried blood spot specimens) for rescreening. Additionally, mothers of newborns with positive results of free carnitine (CO) and 3-hydroxyisovaleryl-carnitine (C5OH) were recalled for re-examination to rule out maternal origin. If both screens were MS/MS-positive, the newborn was suspected of IEM.

### 2.3 NGS

Blood samples of patients and any participating family members were collected, and genomic DNA was extracted using the QIAamp DNA Mini Kit (Hilden, Germany) following the manufacturer’s protocol. The coding exons of target genes were captured using an Agilent High Sensitivity DNA Kit (Agilent, Santa Clara, CA, USA), and libraries generated from enriched DNA were sequenced using the Illumina NovaSeq 6,000 platform (Illumina Inc., San Diego, CA, USA) in the paired-end mode. The average on-target sequencing depth for exome sequencing was 90X. The sequencing reads were aligned to the human reference genome (UCSC GRCh37/hgl9) using the Burrows-Wheeler Aligner. Variant filtering was performed with the PhenoPro (7) phenotype-scoring algorithm. Detected variants were confirmed by PCR and subjected to direct automated sequencing using a 3500XL Genetic Analyzer (Applied Biosystems) per the manufacturer’s specifications. The variant’s pathogenicity was determined using the criteria established by the American College of Medical Genetics and Genomics (8).

## 3 Results

### 3.1 General results of NBS

**Figure 1:**
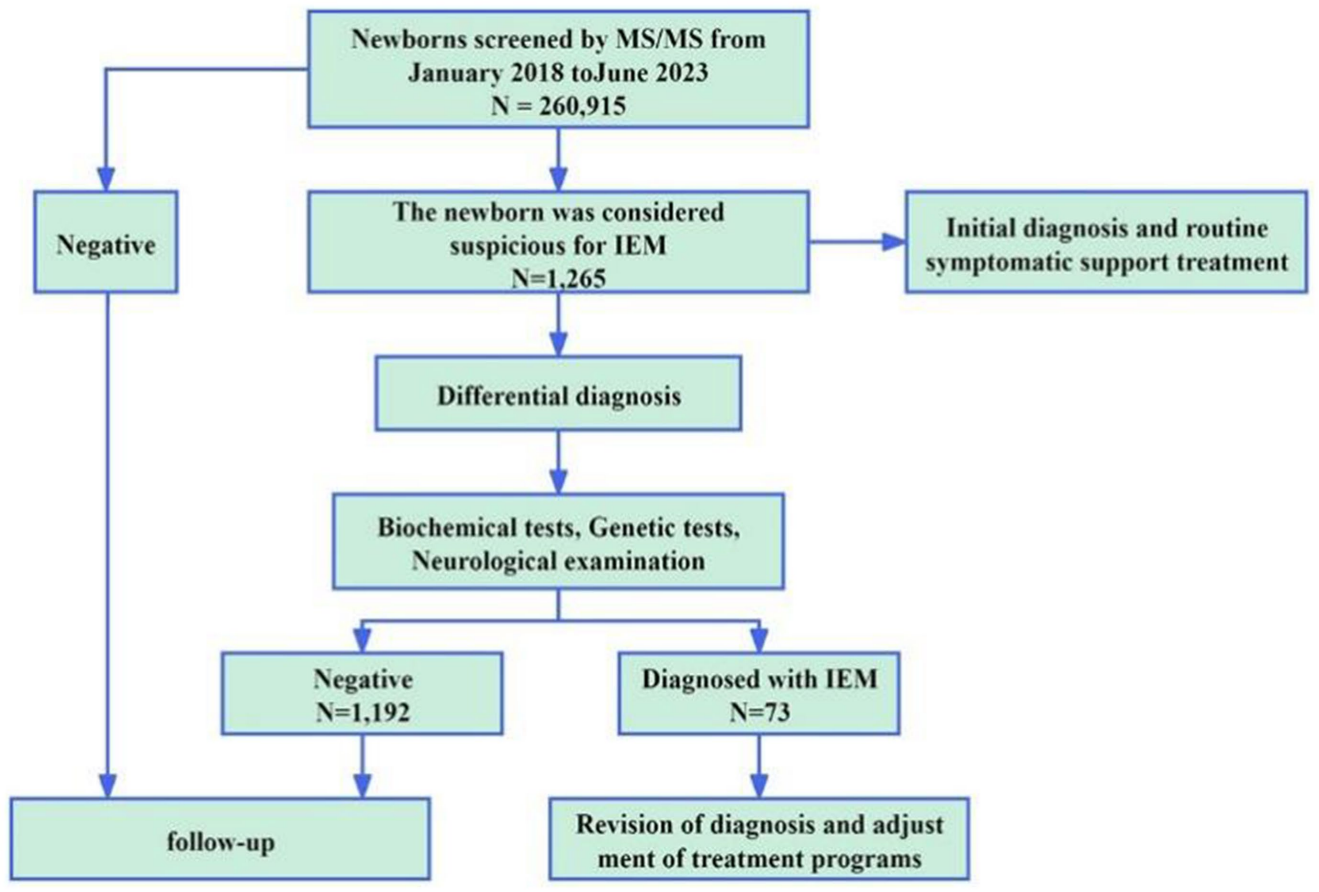
Screening and diagnosis of IEM.

A total of 260,915 newborns underwent MS/MS screening and 1,265 infants (687 male and 578 female) tested positive. The positive results were mainly divided into abnormal amino acid and abnormal acylcarnitine markers. There were amino acid abnormalities, such as increased phenylalanine (Phe) and citrulline (Cit) (15.3 and 9.4% positivity rates, respectively), and carnitine abnormalities, such as decreased CO and increased isovaleryl-carnitine (C5) and C5OH (21.8, 9.3, and 8.1% positivity rates, respectively). Additionally, 3.7% of positive infants had simultaneously elevated or reduced indicators for certain amino acids or acylcarnitines. Following clinical and genetic diagnoses, 73 cases of IEM in newborns were diagnosed, with an overall incidence rate of 1 in 3,574 (Table 1). There cases were related to 12 IEM diseases, including 3 cases of fatty acid metabolic disease (39/73, 53.4%), 3 cases of amino acid metabolic disease (23/73, 31.5%), and 6 cases of organic acid metabolic disease (11/73, 15.1%). The highest incidence rate was that of primary carnitine deficiency (PCD, 1/7,248), followed by that of phenylketonuria (PKU, 1/15,348) and citrine deficiency (CD, 1/43,486). Additionally, 5 cases of PCD were confirmed in mothers of newborns. The overall detection rate of IEM screening in the 260,915 newborn screening population was 1/3345. Figure 1 shows the workflow of NBS.

**Table 1:** The incidence and spectrum of 260,915 newborns in the screening program.

Types of diseases	Cases (*n*)	Accounting for patients (%)	Incidence
Fatty acid metabolic disease	39	53.4	
Primary carnitine deficiency	36	49.3	1/7,248
Short-chain acyl-CoA dehydrogenase deficiency	2	2.7	1/130,458
Medium chain acyl CoA dehydrogenase deficiency	1	1.4	1/260,915
Amino acid metabolic disease	23	31.5	
phenylalanine hydroxylase deficiency	13	17.8	1/20,070
Citrin deficiency	6	8.2	1/43,486
Tetrahydrobiopterin deficiency	4	5.5	1/65,229
Organic acid metabolic disease	11	15.1	
3-Methylcrotonyl-CoA carboxylase deficiency	3	4.1	1/86,972
Glutaric acidemia type I	3	4.1	1/86,972
Methylmalonic acidemia	2	2.7	1/130,458
Biotinidase deficiency	1	1.4	1/260,915
Propionic acidemia	1	1.4	1/260,915
Isovaleric acidemia	1	1.4	1/260,915
Total	73	100%	1/3,574

### 3.2 Results of IME genetic diagnosis

Among the 1,265 infants suspected of having IEM, we performed genetic diagnosis utilizing targeted NGS technology. Following genotyping and interpretation, 73 cases were confirmed as IEM (Supplementary Table S1), comprising 46 cases of compound heterozygosity and 27 cases of homozygosity. Additionally, 12 cases were identified as carriers of recessive disorders (Supplementary Table S2). Specifically, among 73 infants with IEM, MS/MS testing suggested some forms of IEM in 49 cases (49/73, 67.1%). The result in P59 via NGS was inconsistent with that of MS/MS. The results of NGS revealed the homozygosity of *SLC25A13* c.851_854del (p.M285Pfs), classified as CD. In the remaining 48 infants (48/73, 65.8%), the genetic result was consistent with that of MS/MS. Additionally, MS/MS revealed that 24 infants (24/73, 32.9%) suffered from certain kinds of IEM, and then disease types were identified by NGS. Among 24 infants, 17 (P40–P56) showed an increase in Phe and Phe/Tyr by MS/MS detection and 13 were cases of phenylalanine hydroxylase deficiency, while four were cases of tetrahydrobiopterin deficiency by NGS. Three infants (P70–P72) showed an increase in C3 and C3/ C2 by MS/MS detection and two were cases of methylmalonic acidemia and one was a case of propionic acidemia by NGS. Four infants (P63-P66) were detected by MS/MS with C_5_OH increasing; one was a case of biotinidase deficiency and three were cases of 3-Methylcrotonyl-CoA carboxylase deficiency by (3MCC) NGS. Results from confirmatory biochemical tests were employed to verify the genetic findings (Supplementary Table S3). The consistency was observed in the outcomes of 73 cases of genetically confirmed IEM abnormalities. Overall, 72 true positive cases and one false negative case were identified through NGS, and 94.2% (1,192/1,265) of the false positivity results were excluded (Supplementary Table S4).

### 3.3 Analysis of genetic variation

Among 1,265 infants with suspected IEM, 76 variants involving 18 IEM-related genes were detected by NGS. Approximately 64.47% (49/76) of the variants were classified as pathogenic, 10.53% (8/76) were likely pathogenic, and 18.42% (14/76) were categorized as being of uncertain significance, based on the ACMG guidelines and criteria (Supplementary Table S5). The annotation results indicated that 71.1% (54/76) were missense variants, 7.9% (6/76) were frameshift variants, 10.5% (8/76) were splice variants, 6.6% (5/76) were nonsense variants, and 3.9% (3/76) were inframe variants. Additionally, 7.89% (6/76) were novel variants which has not yet been included in the Human Gene Mutation Database, the 1,000 Genomes Project and the Exome Aggregation in the Consortium. These six novel variants were located in four genes, including c.547G > T (p.E183Ter) and c.948delA (p.E316Ter) in *PAH* (NM_000277.1) associated with PKU, c.628G > A (p.E228K) and c.79A > C (p.T27P) in *ACADS* (NM_000017.4) causing short-chain acyl-CoA dehydrogenase deficiency, c.1364G > C (p.R455P) in *SLC25A13* (NM_014251.3) causing CD, and c.493A > C (p.T165P) in *MCCC1* (NM_020166.5) causing 3MCC. These results broadened our understanding of the IEM diseases. Additionally, our findings revealed that variants of the *SLC22A5* gene were the most prevalent, accounting for 41% (77/188) of all identified variants. In 40 cases of primary carnitine, c.51C > G in NM_003060.4 is one of the most common variant, accounting for 36.4% of all variants (28/77) and affecting 55% (22/40) of patients, followed by c.1400C > G (17/77, 22.1% and 17/40, 42.5%). Additionally, variants of the *PAH* gene were also common, accounting for 18.1% (34/188). The most common variant was c.728G > A in NM_000277.3, accounting for 26.5% (9/34) of all variants and affecting 41.2% (7/17) of patients, followed by c.611A > G (5/34, 14.7% and 4/17, 23.5%). Additionally, variants of *SLC25A13* were also common, accounting for 8.0% (15/188). The most common variant was c.852_855del in NM_014251.3, which accounted for 66.7% (10/15) of all variants and affecting 66.7% (6/9) of patients. These variants are pathogenic. These results reflected the variant characteristics of IEM diseases in Ganzhou and provided important information for the clinical diagnosis of other samples in the future.

## 4 Discussion

IEM is a group of diseases that affect the growth and development of newborns and children and even lead to death. Their occurrence is associated with genetic defects in the biosynthesis process of the skin, protease, receptor, carrier, and membrane pump, which the body needs to maintain normal metabolism (9). IEM often leads to progressive and irreversible nerve damage and physical and mental disability, posing a major threat to families and society. In this study, 260,915 neonates were screened for IEM using MS/MS technology, and 12 diseases were detected, with an overall incidence rate of 1 in 3,574. Compared with other regions of China such as Taiwan (10) (1/7,030) and Liuzhou (11) (1/3,733), the overall incidence rate is higher. Hence, performing early IEM screening and accurate diagnosis in this area is of particularly importance.

MS/MS has proven to be a reliable method suitable for clinical use, offering many advantages such as high efficiency, sensitivity, and convenience (4). However, biochemical screening has limitations and *in vivo* metabolism is influenced by many factors, leading to the existence of false positives and lowering the positive predictive value. In this study, 1,265 infants were positive in the MS/MS screening, and 73 cases were ultimately diagnosed by NGS, indicating a 94.2% false-positive rate. The simultaneous increase or decrease of several indicators of amino acids or acylcarnitines in the positive results of MS/MS screening can be influenced by various factors such as gestational age at birth, certain diseases, medications, diet and maternal factors, of which can lead to transient or secondary metabolic disorders (5). Per our findings, C5 was common in acylcarnitine-positive indicators in MS/MS; however, only one case of isovaleric acidemia was diagnosed via NGS. False-positive cases should be followed up on. It was reported that there was the presence of isomers in metabolites, including isovalerylcarnitine, tervalerylcarnitine, and 2-methylbutyrylcarnitine, that are difficult to distinguish by MS/MS (12). It is critical to further clarify the nature of the disease, implement targeted therapy, and exclude false alarms to reduce the unnecessary economic, physical, and mental burden on children and their families (13).

In recent years, an increasing number of genetic detection techniques have been employed in the field of NBS (14, 15). Most studies have indicated that the application of targeted NGS technology advanced NBS diagnosis and treatment and reduced the diagnostic delay (16). In this study, we designed an NGS-based genetic diagnostic panel for IEM. All children underwent identification using the NGS panel and received a definitive diagnosis. Among the identified IEM cases, PCD was the most frequently diagnosed, accounting for 49.3% (36/73) of the total. PCD, also known as the carnitine transport disorder or carnitine uptake disorder, is a fatty acid beta-oxidation disorder resulting from a variant of *SLC22A5* that encodes the carnitine transporter OCTN2 located in the cell membrane (17, 18). PCD is an autosomal recessive inherited disease with an incidence of approximately 1/300–142,000, varying across different countries and ethnic groups (19). The incidence of PCD in NBS in this area is 1/7,248, making it one of the highest incidence areas in China, comparable to Liuzhou (11), Quanzhou (20), and other areas. The most reliable and rapid method for early PCD diagnosis is the MS/MS detection of the CO level (21). However, the CO results of MS/MS screening can be affected by various factors, including the maternal CO level, prematurity, and inadequate intake, and other fatty acid oxidation defects (22). In this study, we employed targeted NGS to advance the differential diagnosis of children with suspected PCD, thereby eliminating false positives resulting from these factors. Several pathogenic variants of *SLC22A5* (NM_003060.4) were found, including c.51C > G, c.1400C > G, c.428C > T, c.338G > A, and c.760C > T, with c.51C > G and c.1400C > G having the highest frequency. It has been reported that c.760C > T (p.R254X) and c.1400C > G (p.S467C) are the two most common variants in patients with PCD (20). However, in this study, the main variants observed were c.51C > G (p.F17L) and c.1400C > G (p.S467C). Previous studies have reported a low variant frequency of c.760C > T (p.R254X) in asymptomatic neonates (23). This finding is consistent with the results of the present study.

PKU is the most common disease of abnormal amino acid metabolism and has the second-highest incidence in this study (1/15,348). This incidence is close to the prevalence rate of live births (1/15,924) in China (24). The incidence of PKU varies considerably between geographical regions, with China having the highest incidence among Asian countries (25). Its pathogenesis is associated with the variants in *PAH,* which encodes the phenylalanine lightening enzyme (26). If patients are not treated promptly, severe and irreversible mental impairment, growth retardation, psychological behavior, acquired microcephaly, systemic skin hypopigmentation, and musty sweat odor may occur (27). The use of MS/MS to detect the Phe concentration and the Phe/Tyr ratio in newborns enable the early detection of PKU in children. However, this method cannot distinguish between different phenotypes; therefore, it may not be suitable for timely and appropriate treatment (28). Thus, the key to treating PKU lies in further clarifying the exact type of PKU. This study demonstrates the effectiveness of targeted NGS technology in eliminating false positives in MS/MS screening and identifying the PKU type and genotype. This enables accurate targeted therapy for infants with specific types of PKU. Consistent with previous reports, early diagnosis and treatment contributed to favorable outcomes for patients with PKU (29). There is a high degree of variability in *PAH* (NM_000277.3), as two of the first variants found were c.1315 + 1G > A and c.1222C > T (p.Arg408Trp) (30). Within a few years, many new variants were discovered, and two of these new variants c.547G > T (p.E183Ter) and c.948delA (p.E316Ter) were also found in this study. According to ACMG and the available evidence, these new variants were classified as pathogenic. To date, over 800 variants in *PAH* have been identified, encompassing more than 100 different types of variants in children with PKU in China (31). It is noteworthy that there were variations in hotspot variants in *PAH* among different regions and populations. According to the results of large-sample research conducted in mainland China (26), the most common variant sites included c.728G > A, c.611A > G, c.331C > T, c.1238G > C, and c.442-1G > A, with c.728G > A having the highest variant frequency. The variant characteristics of *PAH* in this study were consistent with the results of large-cohort studies conducted in mainland China, including eastern China (18) and Nanjing (32). However, the hotspot variant c.158G > A detected in this study is uncertain and requires further validation.

CD, which is inherited in an autosomal recessive manner, is the most common disorder of the urea cycle. It is caused by pathogenic variants of *SLC25A13* (33) and results in a broad spectrum of phenotypes ranging from life-threatening hyperammonemia in neonates to adult-onset hyperammonemia with mild symptoms or no manifestations at all. The detection of neonatal blood amino acids (Cit, Cit/Arg) by MS/MS has a high sensitivity for the early diagnosis of CD children. However, an increasing number of case reports have found that the clinical manifestations and laboratory abnormalities of CD patients are varied and transient (34). This study identified six cases of CD through targeted NGS. One case was detected due to abnormal levels of CO, while the initial results for Cit or Cit/Arg were within the normal range. Therefore, while highly biochemical indicators are not strictly necessary, a combination of clinical manifestations and genetic analyses is essential for making an accurate diagnosis (35). With an incidence rate of 1/43,486, it ranked third in our study. A previous study indicated that the incidence rate in southern China is significantly higher than that in northern China, with provinces at lower latitudes having significantly higher incidence rates than those at higher latitudes (36). In *SLC25A13* (NM_014251.3), c.852_855del, c.1638_1660dup, c.615 + 5G > A, and c.1751-5_1751-4ins were the most common variants in China, accounting for 82.9% of all variants (37). In our study, we observed that c.852_855del was the most prevalent, accounting for 66.7% of cases. These findings are in line with results of previous studies (38).

Although targeted NGS technology has demonstrated many advantages in clinical applications, its high cost compared with MS/MS technology may limit its widespread use in resource-limited areas (39). Furthermore, targeted NGS primarily focuses on detecting known potential targets and exhibits limitations when addressing complex genomic variants, such as structural and copy number variations (40). This limitation could lead to the omission of certain disease-associated variants, potentially impacting clinical decision-making.

## 5 Conclusion

In summary, targeted NGS technology can serve as a crucial diagnostic tool for neonatal genetic metabolic diseases. Its combination with MS/MS technology proves effective and suitable for clinical screening and diagnosis. Additionally, we identified IEM variant hotspots and some novel variants in our region. These variants are the cause of IEM in certain patients, helping to elucidate the etiology of the disease at the genetic level.

## Data Availability

The original contributions presented in the study are included in the article/Supplementary material, further inquiries can be directed to the corresponding author.

## References

[1] Smith LD Willig LK Kingsmore SF. Whole-exome sequencing and whole-genome sequencing in critically ill neonates suspected to have single-gene disorders. Cold Spring Harb Perspect Med. (2015) 6:a023168. doi: 10.1101/cshperspect.a023168, PMID: 26684335 PMC4743073

[2] Guthrie R Susi A. A simple phenylalanine method for detecting phenylketonuria in large populations of newborn infants. Am Acad Pediatr. (1963) 32:338–43. doi: 10.1542/peds.32.3.338, PMID: 14063511

[3] Anetor JI Orimadegun BE Anetor GO. A pragmatic approach to the diagnosis of inborn errors of metabolism in developing countries. Afr J Lab Med. (2023) 12:1946. doi: 10.4102/ajlm.v12i1.1946, PMID: 37293316 PMC10244816

[4] Demirelce Ö Aksungar FB Saral NY Kilercik M Serteser M Unsal I. Institutional experience of newborn screening for inborn metabolism disorders by tandem MS in the Turkish population. J Pediatr Endocrinol Metab. (2020) 33:703–11. doi: 10.1515/jpem-2019-0571, PMID: 32469332

[5] Liu J Chen XX Li XW Fu W Zhang WQ. Metabolomic research on newborn infants with intrauterine growth restriction. Medicine. (2016) 95:e3564. doi: 10.1097/md.0000000000003564, PMID: 27124067 PMC4998730

[6] Wang X Wang YY Hong DY Zhang ZL Li YH Yang PY. Combined genetic screening and traditional biochemical screening to optimize newborn screening systems. Clin Chim Acta. (2022) 528:44–51. doi: 10.1016/j.cca.2022.01.015, PMID: 35085585

[7] Li Z Zhang F Wang Y Qiu Y Wu Y Lu Y. PhenoPro: a novel toolkit for assisting in the diagnosis of Mendelian disease. Bioinformatics. (2019) 35:3559–66. doi: 10.1093/bioinformatics/btz100, PMID: 30843052

[8] Richards S Aziz N Bale S Bick D Das S Gastier-Foster J. Standards and guidelines for the interpretation of sequence variants: a joint consensus recommendation of the American College of Medical Genetics and Genomics and the Association for Molecular Pathology. Genet Med. (2015) 17:405–24. doi: 10.1038/gim.2015.30, PMID: 25741868 PMC4544753

[9] Yunus ZM Rahman SA Choy YS Keng WT Ngu LH. Pilot study of newborn screening of inborn error of metabolism using tandem mass spectrometry in Malaysia: outcome and challenges. J Pediatr Endocrinol Metab. (2016) 29:1031–9. doi: 10.1515/jpem-2016-0028, PMID: 27544719

[10] Shibata N Hasegawa Y Yamada K Kobayashi H Purevsuren J Yang Y. Diversity in the incidence and spectrum of organic acidemias, fatty acid oxidation disorders, and amino acid disorders in Asian countries: selective screening vs. expanded newborn screening. Mol Genet Metab Rep. (2018) 16:5–10. doi: 10.1016/j.ymgmr.2018.05.003, PMID: 29946514 PMC6014585

[11] Tan J Chen D Chang R Pan L Yang J Yuan D. Tandem mass spectrometry screening for inborn errors of metabolism in newborns and high-risk infants in southern China: disease Spectrum and genetic characteristics in a Chinese population. Front Genet. (2021) 12:631688. doi: 10.3389/fgene.2021.631688, PMID: 34394177 PMC8355895

[12] Minkler PE Stoll MSK Ingalls ST Hoppel CL. Selective and accurate C5 acylcarnitine quantitation by UHPLC-MS/MS: distinguishing true isovaleric acidemia from pivalate derived interference. J Chromatogr B Analyt Technol Biomed Life Sci. (2017) 1061-1062:128–33. doi: 10.1016/j.jchromb.2017.07.018, PMID: 28734160

[13] Tu WJ He J Chen H Shi XD Li Y. Psychological effects of false-positive results in expanded newborn screening in China. PLoS One. (2012) 7:e36235. doi: 10.1371/journal.pone.0036235, PMID: 22558398 PMC3338668

[14] Roman TS Crowley SB Roche MI Foreman AKM O'Daniel JM Seifert BA. Genomic sequencing for newborn screening: results of the NC NEXUS project. Am J Hum Genet. (2020) 107:596–611. doi: 10.1016/j.ajhg.2020.08.001, PMID: 32853555 PMC7536575

[15] Yang RL Qian GL Wu DW Miao JK Yang X Wu BQ. A multicenter prospective study of next-generation sequencing-based newborn screening for monogenic genetic diseases in China. World J Pediatr. (2023) 19:663–73. doi: 10.1007/s12519-022-00670-x, PMID: 36847978 PMC10258179

[16] van Campen JC Sollars ESA Thomas RC Bartlett CM Milano A Parker MD. Next generation sequencing in newborn screening in the United Kingdom National Health Service. Int J Neonatal Screen. (2019) 5:40. doi: 10.3390/ijns5040040, PMID: 31844782 PMC6914376

[17] Crefcoeur LL Visser G Ferdinandusse S Wijburg FA Langeveld M Sjouke B. Clinical characteristics of primary carnitine deficiency: a structured review using a case-by-case approach. J Inherit Metab Dis. (2022) 45:386–405. doi: 10.1002/jimd.12475, PMID: 34997761 PMC9305179

[18] Men S Liu S Zheng Q Yang S Mao H Wang Z. Incidence and genetic variants of inborn errors of metabolism identified through newborn screening: a 7-year study in eastern coastal areas of China. Mol Genet Genomic Med. (2023) 11:e2152. doi: 10.1002/mgg3.2152, PMID: 36787440 PMC10265071

[19] Lefèvre CR Labarthe F Dufour D Moreau C Faoucher M Rollier P. Newborn screening of primary carnitine deficiency: an overview of worldwide practices and pitfalls to define an algorithm before expansion of newborn screening in France. Int J Neonatal Screen. (2023) 9:6. doi: 10.3390/ijns9010006, PMID: 36810318 PMC9944086

[20] Lin W Wang K Zheng Z Chen Y Fu C Lin Y. Newborn screening for primary carnitine deficiency in Quanzhou, China. Clin Chim Acta. (2021) 512:166–71. doi: 10.1016/j.cca.2020.11.005, PMID: 33181153

[21] Chang S Yang Y Xu F Ji W Zhan X Gao X. Clinical, biochemical, and molecular genetic characteristics of patients with primary carnitine deficiency identified by newborn screening in Shanghai, China. Front Genet. (2022) 13:1062715. doi: 10.3389/fgene.2022.1062715, PMID: 36568374 PMC9772520

[22] Lin Y Xu H Zhou D Hu Z Zhang C Hu L. Screening 3.4 million newborns for primary carnitine deficiency in Zhejiang Province, China. Clin Chim Acta. (2020) 507:199–204. doi: 10.1016/j.cca.2020.04.039, PMID: 32371215

[23] Han L Wang F Wang Y Ye J Qiu W Zhang H. Analysis of genetic mutations in Chinese patients with systemic primary carnitine deficiency. Eur J Med Genet. (2014) 57:571–5. doi: 10.1016/j.ejmg.2014.08.001, PMID: 25132046

[24] Hillert A Anikster Y Belanger-Quintana A Burlina A Burton BK Carducci C. The genetic landscape and epidemiology of phenylketonuria. Am J Hum Genet. (2020) 107:234–50. doi: 10.1016/j.ajhg.2020.06.006, PMID: 32668217 PMC7413859

[25] Xiang L Tao J Deng K Li X Li Q Yuan X. Phenylketonuria incidence in China between 2013 and 2017 based on data from the Chinese newborn screening information system: a descriptive study. BMJ Open. (2019) 9:e031474. doi: 10.1136/bmjopen-2019-031474, PMID: 31444193 PMC6707664

[26] Li N Jia H Liu Z Tao J Chen S Li X. Molecular characterisation of phenylketonuria in a Chinese mainland population using next-generation sequencing. Sci Rep. (2015) 5:15769. doi: 10.1038/srep15769, PMID: 26503515 PMC4621502

[27] de Almeida BNF Laufer JA Mezzomo TR Shimada NC Furtado IHF Dias M. Nutritional and metabolic parameters of children and adolescents with phenylketonuria. Clin Nutr ESPEN. (2020) 37:44–9. doi: 10.1016/j.clnesp.2020.03.024, PMID: 32359754

[28] Tendi EA Guarnaccia M Morello G Cavallaro S. The utility of genomic testing for Hyperphenylalaninemia. J Clin Med. (2022) 11:1061. doi: 10.3390/jcm11041061, PMID: 35207333 PMC8879487

[29] Maissen-Abgottspon S Muri R Hochuli M Reismann P Barta AG Alptekin IM. Health-related quality of life in a european sample of adults with early-treated classical PKU. Orphanet J Rare Dis. (2023) 18:300. doi: 10.1186/s13023-023-02917-w, PMID: 37740225 PMC10517574

[30] Gable M Williams M Stephenson A Okano Y Ring S Hurtubise M. Comparative multiplex dosage analysis detects whole exon deletions at the phenylalanine hydroxylase locus. Hum Mutat. (2003) 21:379–86. doi: 10.1002/humu.10199, PMID: 12655547

[31] Zhang Z Gao JJ Feng Y Zhu LL Yan H Shi XF. Mutational spectrum of the phenylalanine hydroxylase gene in patients with phenylketonuria in the central region of China. Scand J Clin Lab Invest. (2018) 78:211–8. doi: 10.1080/00365513.2018.1434898, PMID: 29390883

[32] Wang X Wang Y Ma D Zhang Z Li Y Yang P. Neonatal screening and genotype-phenotype correlation of hyperphenylalaninemia in the Chinese population. Orphanet J Rare Dis. (2021) 16:214. doi: 10.1186/s13023-021-01846-w, PMID: 33980295 PMC8114530

[33] Kobayashi K Sinasac DS Iijima M Boright AP Begum L Lee JR. The gene mutated in adult-onset type II citrullinaemia encodes a putative mitochondrial carrier protein. Nat Genet. (1999) 22:159–63. doi: 10.1038/9667, PMID: 10369257

[34] Lipiński P Jurkiewicz D Ciara E Płoski R Więcek S Bogdańska A. Neonatal cholestasis due to citrin deficiency: diagnostic pitfalls. Acta Biochim Pol. (2020) 67:225–8. doi: 10.18388/abp.2020_5202, PMID: 32436673

[35] Chen HA Hsu RH Chen YH Hsu LW Chiang SC Lee NC. Improved diagnosis of citrin deficiency by newborn screening using a molecular second-tier test. Mol Genet Metab. (2022) 136:330–6. doi: 10.1016/j.ymgme.2022.06.007, PMID: 35798653

[36] Zhao B Chen P She X Chen X Ni Z Zhou D. China nationwide landscape of 16 types inherited metabolic disorders: a retrospective analysis on 372,255 clinical cases. Orphanet J Rare Dis. (2023) 18:228. doi: 10.1186/s13023-023-02834-y, PMID: 37537594 PMC10398906

[37] Lin WX Zeng HS Zhang ZH Mao M Zheng QQ Zhao ST. Molecular diagnosis of pediatric patients with citrin deficiency in China: SLC25A13 mutation spectrum and the geographic distribution. Sci Rep. (2016) 6:29732. doi: 10.1038/srep29732, PMID: 27405544 PMC4942605

[38] Lin WX Yaqub MR Zhang ZH Mao M Zeng HS Chen FP. Molecular epidemiologic study of citrin deficiency by screening for four reported pathogenic SLC25A13 variants in the Shaanxi and Guangdong provinces. Transl Pediatr. (2021) 10:1658–67. doi: 10.21037/tp-21-58 34295780 PMC8261583

[39] Howard HC Knoppers BM Cornel MC Wright Clayton E Sénécal K Borry P. Whole-genome sequencing in newborn screening? A statement on the continued importance of targeted approaches in newborn screening programmes. Eur J Hum Genet. (2015) 23:1593–600. doi: 10.1038/ejhg.2014.289, PMID: 25626707 PMC4795188

[40] Platt CD Zaman F Bainter W Stafstrom K Almutairi A Reigle M. Efficacy and economics of targeted panel versus whole-exome sequencing in 878 patients with suspected primary immunodeficiency. J Allergy Clin Immunol. (2021) 147:723–6. doi: 10.1016/j.jaci.2020.08.022, PMID: 32888943 PMC7870529

