# Supplementary material for: Application of targeted high-throughput sequencing as a diagnostic tool for neonatal genetic metabolic diseases following tandem mass spectrometry screening
Source: Front Public Health. 2024 Dec 24;12:1461141. doi: 10.3389/fpubh.2024.1461141 (PMC11703805; doi:10.3389/fpubh.2024.1461141)
Supplement: Supplementary file 1 [file Table_1.DOCX]

Supplementary Table S1 Summary of clinical and genetic features of 73 diagnosed infants by NGS.

| Case | Sex | GA  (weeks) | BW (g) | Gene | Variant allele 1 | Variant allele 2 | MS/MS  Results (µmol/L) | Disorders |
| --- | --- | --- | --- | --- | --- | --- | --- | --- |
| P1 | F | 38 | 2600 | *SLC22A5* | c.1400C>G(p.S467C) | c.428C>T(p.P143L) | C0=7.34 | PCD |
| P2 | M | 38+4 | 2700 | *SLC22A5* | c.338G>A(p.C113Y) | c.428C>T(p.P143L) | C0=4.94 | PCD |
| P3 | F | 38+4 | 2750 | *SLC22A5* | c.338G>A(p.C113Y) | c.428C>T(p.P143L) | C0=5.18 | PCD |
| P4 | M | 40 | 2700 | *SLC22A5* | c.51C>G(p.F17L) | c.1400C>G(p.S467C) | C0=6.58 | PCD |
| P5 | F | 38+1 | 3400 | *SLC22A5* | c.51C>G(p.F17L) | c.1400C>G(p.S467C) | C0=7.66 | PCD |
| P6 | F | 40 | 3600 | *SLC22A5* | c.338G>A(p.C113Y) | c.338G>A(p.C113Y) | C0=1.55 | PCD |
| P7 | M | 37+4 | 2800 | *SLC22A5* | c.760C>T(p.R254Ter) | c.1400C>G(p.S467C) | C0=4.93 | PCD |
| P8 | F | 39+2 | 3200 | *SLC22A5* | c.51C>G(p.F17L) | c.1400C>G(p.S467C) | C0=6.77 | PCD |
| P9 | M | 39+5 | 3700 | *SLC22A5* | c.1195C>T(p.R399W) | c.1400C>G(p.S467C) | C0=7.25 | PCD |
| P10 | M | 40 | 3100 | *SLC22A5* | c.51C>G(p.F17L) | c.1400C>G(p.S467C) | C0=5.58 | PCD |
| P11 | M | 38+3 | 3600 | *SLC22A5* | c.51C>G(p.F17L) | c.51C>G(p.F17L) | C0=4.84 | PCD |
| P12 | M | 37+4 | 3300 | *SLC22A5* | c.51C>G(p.F17L) | c.1400C>G(p.S467C) | C0=2.62 | PCD |
| P13 | F | 41 | 3500 | *SLC22A5* | c.428C>T(p.P143L) | c.428C>T(p.P143L) | C0=2.82 | PCD |
| P14 | F | 39+3 | 3350 | *SLC22A5* | c.338G>A(p.C113Y) | c.1400C>G(p.S467C) | C0=3.75 | PCD |
| P15 | F | 39 | 3100 | *SLC22A5* | c.51C>G(p.F17L) | c.1400C>G(p.S467C) | C0=4.87 | PCD |
| P16 | F | 40+2 | 3400 | *SLC22A5* | c.51C>G(p.F17L) | c.428C>T(p.P143L) | C0=4.73 | PCD |
| P17 | M | 41 | 3370 | *SLC22A5* | c.51C>G(p.F17L) | c.760C>T(p.R254Ter) | C0=5.49 | PCD |
| P18 | M | 39+6 | 3400 | *SLC22A5* | c.51C>G(p.F17L) | c.51C>G(p.F17L) | C0=3.51 | PCD |
| P19 | F | 40+4 | 3100 | *SLC22A5* | c.51C>G(p.F17L) | c.51C>G(p.F17L) | C0=7.24 | PCD |
| P20 | M | 39+2 | 3500 | *SLC22A5* | c.1195C>T(p.R399W) | c.1400C>G(p.S467C) | C0=3.48 | PCD |
| P21 | M | 40 | 3580 | *SLC22A5* | c.51C>G(p.F17L) | c.760C>T(p.R254Ter) | C0=4.49 | PCD |
| P22 | M | 40+3 | 3200 | *SLC22A5* | c.338G>A(p.C113Y) | c.1400C>G(p.S467C) | C0=5.19 | PCD |
| P23 | M | 38+5 | 3330 | *SLC22A5* | c.51C>G(p.F17L) | c.51C>G(p.F17L) | C0=5.13 | PCD |
| P24 | F | 39 | 3550 | *SLC22A5* | c.51C>G(p.F17L) | c.338G>A(p.C113Y) | C0=1.38 | PCD |
| P25 | M | 40 | 3100 | *SLC22A5* | c.1400C>G(p.S467C) | c.428C>T(p.P143L) | C0=6.29 | PCD |
| P26 | F | 39 | 3300 | *SLC22A5* | c.428C>T(p.P143L) | c.1400C>G(p.S467C) | C0=5.13 | PCD |
| P27 | F | 40 | 3000 | *SLC22A5* | c.760C>T(p.R254Ter) | c.1400C>G(p.S467C) | C0=2.71 | PCD |
| P28 | F | 40 | 3350 | *SLC22A5* | c.51C>G(p.F17L) | c.51C>G(p.F17L) | C0=2.91 | PCD |
| P29 | F | 40 | 3750 | *SLC22A5* | c.51C>G(p.F17L) | c.51C>G(p.F17L) | C0=2.76 | PCD |
| P30 | M | 38 | 3400 | *SLC22A5* | c.51C>G(p.F17L) | c.1400C>G(p.S467C) | C0=2.95 | PCD |
| P31 | F | 38 | 3500 | *SLC22A5* | c.338G>A(p.C113Y) | c.338G>A(p.C113Y) | C0=0.6 | PCD |
| P32 | M | 40 | 3700 | *SLC22A5* | c.51C>G(p.F17L) | c.797C>T(p.P266L) | C0=8.38 | PCD |
| P33 | M | 39 | 2700 | *SLC22A5* | c.51C>G(p.F17L) | c.338G>A(p.C113Y) | C0=4.12 | PCD |
| P34 | M | 38 | 2500 | *SLC22A5* | c.428C>T(p.P143L) | c.1400C>G(p.S467C) | C0=6.72 | PCD |
| P35 | M | 39 | 1900 | *SLC22A5* | c.51C>G(p.F17L) | c.1412G>A(p.R471H) | C0=7.42 | PCD |
| P36 | F | 42 | 4500 | *SLC22A5* | c.51C>G(p.F17L) | c.51C>G(p.F17L) | C0=3.76 | PCD |
| P37 | F | 39 | 3700 | *ACADS* | c.1031A>G(p.E344G) | c.1031A>G(p.E344G) | C4=1.67, C4/C3=1.3 | SCAD |
| P38 | M | 38 | 3200 | *ACADS* | c.319C>T(p.R107C) | c.988C>T(p.R330C) | C4=1.9, C4/C3=1.21 | SCAD |
| P39 | M | 38+2 | 3000 | *ACADM* | c.424_426del  (p.Lys144del) | c.668T>C(p.I223T) | C8=1.88, C8/C3=0.35  C8/C10=13.43 | MCAD |
| P40 | F | 40+3 | 3550 | *PAH* | c.907delT  (p.S303PfsTer38) | **c.948delA**  **(p.E316Ter)** | Phe=2160  Phe/Tyr=27.8 | PAHD |
| P41 | M | 39+6 | 3850 | *PAH* | **c.547G>T(p.E183Ter)** | c.1174T>A(p.F392I) | Phe=267, Phe/Tyr=2.57 | PAHD |
| P42 | M | 38+4 | 3800 | *PAH* | c.611A>G(p.Y204C) | c.168+1G>A | Phe=764, Phe/Tyr=21.3 | PAHD |
| P43 | F | 39+5 | 2950 | *PAH* | c.782G>A(p.R261E) | c.707-1G>A | Phe=635.2, Phe/Tyr=11.9 | PAHD |
| P44 | F | 39 | 3650 | *PAH* | c.754C>T(p.R252W) | c.754C>T(p.R252W) | Phe=474, Phe/Tyr=13.6 | PAHD |
| P45 | F | 39 | 3800 | *PAH* | c.611A>G  (p.Y204C) | c.611A>G  (p.Y204C)) | Phe=852.95, Phe/Tyr=7.55 | PAHD |
| P46 | F | 41 | 3200 | *PAH* | c.842+1G>A  (p.?/IVS7+1G>A) | c.722delG (p.R241fs ) | Phe=821.58  Phe/Tyr=14.38 | PAHD |
| P47 | F | 39 | 3000 | *PAH* | c.728G>A(p.R243Q) | c.728G>A(p.R243Q) | Phe=232, Phe/Tyr=3.48 | PAHD |
| P48 | M | 41 | 3400 | *PAH* | c.251A>G(p.D84G) | c.617A>G(p.Y206C) | Phe=423, Phe/Tyr=4.09 | PAHD |
| P49 | M | 33 | 1900 | *PAH* | c.728G>A(p.R243Q) | c.728G>A(p.R243Q) | Phe=2410, Phe/Tyr=28.4 | PAHD |
| P50 | M | 39 | 2600 | *PAH* | c.875C>T(p.P292L) | c.728G>A(p.R243Q) | Phe=2059, Phe/Tyr=27 | PAHD |
| P51 | M | 36 | 2750 | *PAH* | c.611A>G(p.Y204C) | c.728G>A(p.R243Q) | Phe=836, Phe/Tyr=1.29 | PAHD |
| P52 | F | 41 | 2300 | *PAH* | c.611A>G(p.Y204C) | c.728G>A(p.R243Q) | Phe=765, Phe/Tyr=1.88 | PAHD |
| P53 | F | 31+4 | 1430 | *PTS* | c.155A>G(p.N52S) | c.155A>G(p.N52S) | Phe=634, Phe/Tyr=10.63 | BH4D |
| P54 | F | 38 | 3700 | *PTS* | c.259C>T(p.P87S) | c.331G>A (p.A111T) | Phe=345.28, Phe/Tyr=6.3 | BH4D |
| P55 | F | 40+4 | 3450 | *PTS* | c.259C>T(p.P87S) | c.286G>A(p.D96N) | Phe=1972, Phe/Tyr=21.1 | BH4D |
| P56 | M | 39+5 | 3400 | *PTS* | c.259C>T(p.P87S) | c.259C>T( p.P87S ) | Phe=513, Phe/Tyr=9.13 | BH4D |
| P57 | M | 39+1 | 2500 | *SLC25A13* | c.1067G>A(p.R356Q) | c.852_855del  (p.M285PfsTer2) | Cit=171.34, Cit/Phe=0.69 | CD |
| P58 | M | 39 | 1900 | *SLC25A13* | c.852_855del  (p.M285PfsTer2) | c.852_855del  (p.M285PfsTer2) | Cit=284.15, Cit/Phe=3.46 | CD |
| P59 | M | 38+4 | 3000 | *SLC25A13* | c.851_854del  (p.M285Pfs) | c.851_854del  (p.M285Pfs) | C0=8.62 | CD |
| P60 | F | 38+3 | 3000 | *SLC25A13* | c.852_855del  (p.M285PfsTer2) | c.852_855del  (p.M285PfsTer2) | Cit=400.15, Cit/Phe=9.27 | CD |
| P61 | F | 39 | 2069 | *SLC25A13* | c.852_855del  (p.M285PfsTer2) | c.852_855del  (p.M285PfsTer2) | Cit=301.45, Cit/Phe=2.33 | CD |
| P62 | M | 40 | 3600 | *SLC25A13* | c.852_855del  (p.M285PfsTer2) | c.852_855del  (p.M285PfsTer2) | Cit=255, Cit/Phe=3.93 | CD |
| P63 | M | 40+6 | 3120 | *MCCC1* | c.639+2T>A  (IVS6dsT-A+2) | c.639+2T>A  (IVS6dsT-A+2) | C5OH=4.37 C5OH/C8=109 | 3MCC |
| P64 | M | 38 | 3300 | *MCCC1* | c.639+2T>A  (IVS6dsT-A+2) | c.863A>G(p.E288G) | C5OH=3.42 C5OH/C8=171 | 3MCC |
| P65 | M | 39+4 | 3400 | *MCCC2* | c.351_353del  (p.G118del) | c.538C>T(p.R180Ter) | C5OH=11.68, C5OH/C8=389.33 | 3MCC |
| P66 | F | 40+3 | 2950 | *BTD* | c.1211G>C(p.C404S) | c.1211G>C(p.C404S) | C5OH=1.24 | BTDD |
| P67 | M | 39+6 | 3450 | *GCDH* | c.700C>T(p.R234W) | c.1045G>A(p.A349T) | C5DC=2.65 C5DC/C8=132.5 | GA-Ι |
| P68 | F | 39 | 3200 | *GCDH* | c.1244-2A>G | c.1244-2A>G | C5DC=2.95 C5DC/C8=73.75 | GA-Ι |
| P69 | F | 36 | 2700 | *GCDH* | c.1063C>T(p.R355C) | c.1060G>A(p.G354S) | C5DC=3.99 C5DC/C8=199.5 | GA-Ι |
| P70 | M | 39 | 3600 | *MMAA* | c.742C>T(p.Q248Ter) | c.742C>T(p.Q248Ter) | C3=8.01, C3/C2=0.63 | MMA |
| P71 | M | 40+2 | 3050 | *MMACHC* | c.398_399del  (p.Q133RfsTer5) | c.609G>A(p.W203X) | C3=7.19, C3/C2=0.43 | MMA |
| P72 | F | 39+4 | 2810 | *PCCB* | c.1087T>C(p.S363P) | c.1087T>C(p.S363P) | C3=7.18, C3/C2=0.61 | PA |
| P73 | F | 41 | 3000 | *IVD* | c.148C>T  (p.R50C) | c.1199A>G  (p.Y400C) | C5=12.34, C5/C3=9.95 | IVA |

Abbreviations: PAHD, Phenylalanine hydroxylase deficiency; BH4D, Tetrahydrobiopterin deficiency; CD, Citrin deficiency; MMA, Methylmalonic acidemia; PA, Propionic acidemia; IVA, isovaleric acidemia; GA-Ι, Glutaric acidemia type I; 3MCC, 3-Methylcrotonyl-CoA carboxylase deficiency; BTDD, Biotinidase deficiency; PCD, Primary carnitine deficiency; SCAD, Short-chain acyl-CoA dehydrogenase deficiency; MCAD, Medium chain acyl CoA dehydrogenase deficiency; GA, Gestational age; BW, Birthweight; Phe, phenylalanine; Tyr, tyrosine; Cit, citrulline; C0, free carnitine; C2, acetylcarnitine; C3, propionylcarnitine; C4, butyrylcarnitine; C5, isovalerylcarnitine/2-methylbutyrylcarnitine; C5OH, 3-hydroxy-isovalerylcarnitine; C6, Hexanoylcarnitine; C8, octanoylcarnitine; C5DC, glutarylcarnitine/3-hydroxydecanoylcarnitine; Mutations in bold are novel. Genome reference hg19/GRCh37.
