# Supplementary material for: Application of targeted high-throughput sequencing as a diagnostic tool for neonatal genetic metabolic diseases following tandem mass spectrometry screening
Source: Front Public Health. 2024 Dec 24;12:1461141. doi: 10.3389/fpubh.2024.1461141 (PMC11703805; doi:10.3389/fpubh.2024.1461141)
Supplement: Supplementary file 2 [file Table_2.DOCX]

Supplementary Table S2 12 cases of recessive disease carriers detection by NGS.

| Case | Sex | GA  (weeks) | BW (g) | Gene | Variant allele | | MS/MS  Results (µmol/L) | Disorders |
| --- | --- | --- | --- | --- | --- | --- | --- | --- |
| P74 | F | 39 | 3300 | SLC22A5 | c.1195C>T(p.R399W) | | C0=9.05 | PCD |
| P75 | M | 39 | 3750 | ACADS | c.1031A>G(p.E344G) | | C4=1.79,C4/C3=0.83 | SCAD |
| P76 | M | 40 | 3150 | PAH | c.442-1G>A | | Phe=202,Phe/Tyr=1.6 | PAHD |
| P77 | F | 36 | 3000 | PAH | c.728G>A(p.R243Q) | | Phe=185,Phe/Tyr=1.3 | PAHD |
| P78 | F | 39 | 3700 | PAH | c.1238G>C(p.R413P) | | Phe=243.26,Phe/Tyr=2.23 | PAHD |
| P79 | M | 37 | 3000 | PAH | c.728G>A(p.R243Q) | | Phe=149,Phe/Tyr=1.22 | PAHD |
| P80 | M | 40+2 | 3800 | PTS | c.259C>T(p.P87S) | | Phe=222.06,Phe/Tyr=2.13 | BH4D |
| P81 | F | 39 | 3000 | SLC25A13 | c.615+5G>A | | Cit=51 | CD |
| P82 | M | 37+5 | 2400 | SLC25A13 | c.852_855del(p.M285PfsTer2) | | Met =58.98 | CD |
| P83 | M | 38 | 2700 | MCCC1 | c.196C>T(p.R66C) | | C5OH=0.85,C5OH/C8=21.25 | 3MCC |
| P84 | F | 37+6 | 3250 | MCCC1 | **c.493A>C(p.T165P)** | | C5OH=1.15,C5OH/C8=24.75 | 3MCC |
| P85 | F | 37+1 | 3000 | CPS1 | c.952C>A(p.Q318K) | Cit=4.84 | | CPS-I |

Abbreviations: PAHD, Phenylalanine hydroxylase deficiency; BH4D, Tetrahydrobiopterin deficiency; CD, Citrin deficiency; 3MCC, 3-Methylcrotonyl-CoA carboxylase deficiency; PCD, Primary carnitine deficiency; SCAD, Short-chain acyl-CoA dehydrogenase deficiency; CPS-I, Carnitine palmitoyltransferase I deficiency; BW, Birthweight; Phe, phenylalanine; Tyr, tyrosine; Met, methionine; Cit, citrulline; C0, free carnitine; C4, butyrylcarnitine; C8, octanoylcarnitine; C5OH, 3-hydroxy-isovalerylcarnitine; Genome reference hg19/GRCh37.
