# Supplementary material for: Application of targeted high-throughput sequencing as a diagnostic tool for neonatal genetic metabolic diseases following tandem mass spectrometry screening
Source: Front Public Health. 2024 Dec 24;12:1461141. doi: 10.3389/fpubh.2024.1461141 (PMC11703805; doi:10.3389/fpubh.2024.1461141)
Supplement: Supplementary file 3 [file Table_3.DOC]

**Supplementary Table S3 Biochemical characteristics and genetic diagnosis of 1265 suspected IEM cases**

| LC-MS/MS Main abnormal indicators | Abnormal range values (μmol /L) | Normal reference range (μmol /L) | Number of cases （n=1265） | Possible disorder (s) | Clinical Diagnosis (n=73) | genetic diagnosis (n=73) |  |
| --- | --- | --- | --- | --- | --- | --- | --- |
| Phe | 137.28-2410 | 20.00-120.00 | 193 | Phenylketonuria | 13 cases of phenylalanine hydroxylase deficiency and 4 cases of Tetrahydrobiopterin deficiency | 13 cases of phenylalanine hydroxylase deficiency and 4 cases of Tetrahydrobiopterin deficiency |  |
| Phe/Tyr | 3.6-28.4 | 0.20-1.20 |  |
| Met | 50.40-127.86 | 6.00-40.00 | 65 | Hyperhomocysteinemia; Methionine adenosyltransferase deficiency | 0 | 0 |  |
| Met/Phe | 0.79-2.24 | 0.12-0.70 |  |
| Cit | 36.34-400.15 | 6.50-35.00 | 119 | Citrin deficiency | 5 | 5 |  |
| Cit/Phe | 0.69-9.27 | 0.12-0.65 |  |
| Cit | 2.78-5.8 | 6.50-35.00 | 24 | Ornithine transcarbamylase deficiency; Carbamogl phosphate synthe tose 1 | 0 | 0 |  |
|  |
| Arg | 61-131.3 | 1.4-50 | 31 | Argininemia | 0 | 0 |  |
| Arg/Phe | 1.1-2.35 | 0-1 |  |
| Tyr | 354-603 | 24-307.9 | 18 | Tyrosinemia | 0 | 0 |  |
| Pro | 491-630 | 60-450 | 13 | Hyperprolinemia | 0 | 0 |  |
| C3 | 0.6-8.01 | 0.40-4.20 | 64 | Methylmalonic acidemia; Propionic acidemia | 2 cases of Methylmalonic acidemia and one case of Propionic acidemia | 2 cases of Methylmalonic acidemia and one case of Propionic acidemia |  |
| C3/C2 | 0.3-0.63 | 0.04-0.25 |  |
| C5DC | 0.56-3.99 | 0.04-0.25 | 54 | Glutaric acidemia type I | 3 | 3 |  |
| C5DC/C8 | 38.12-199.5 | 0.50-3 |  |
| C5OH | 0.89-11.68 | 0.08-0.45 | 102 | 3-Hydroxy-3-methylglutaryl CoA lyase deficiency; 3-Methylcrotonyl-CoA carboxylase deficiency; Biotinidase deficiency | 3 cases of 3-Methylcrotonyl-CoA carboxylase deficiency and one case of Biotinidase deficiency. | 3 cases of 3-Methylcrotonyl-CoA carboxylase deficiency and one case of Biotinidase deficiency. |  |
|  |
| C5OH/C8 | 21.25-389.33 | 1.5-15 |  |
| C0 | 5.21-9.62 | 10.00-60.00 | 276 | Primary carnitine deficiency | 36 cases of Primary carnitine deficiency and one case of Citrin deficiency | 36 cases of Primary carnitine deficiency and one case of Citrin deficiency |  |
| C4 | 0.67-2.79 | 0.07-0.45 | 38 | Isobutyryl-CoA dehydrogenase deficiency; Ethylmalonic encephalopathy; Short-chain acyl-CoA dehydrogenase deficiency | 2 cases of Short-chain acyl-CoA dehydrogenase deficiency | 2 cases of Short-chain acyl-CoA dehydrogenase deficiency |  |
|  |
| C4/C3 | 0.27-2.29 | 0.03-0.16 |  |
| C8 | 0.21-1.88 | 0.01-0.17 | 33 | Medium chain acyl CoA dehydrogenase deficiency | 1 | 1 |  |
| C8/C3 | 0.03-0.35 | 0-0.02 |  |
| C8/C10 | 2.12-13.43 | 0.3-1.5 |  |
| C16 | 7.28-11.72 | 0.40-6.00 | 59 | Carnitine palmitoyltransferase II deficiency | 0 | 0 |  |
| C18 | 2.11-2.83 | 0.2-1.8 |  |
| C14 | 0.36-0.45 | 0.04-0.35 |  |
| C10:2 | 0.42-1.83 | 0.04-0.35 | 11 | 2,4-Dienoyl-CoA reductase deficiency | 0 | 0 |  |
| C5 | 0.31-12.34 | 0.03-0.26 | 118 | isovaleric acidemia | 1 | 1 |  |
| C5/C3 | 0.34-9.95 | 0.02-0.3 |  |
| Multiple amino acid or acylcarnitine abnormalities | | | 47 | Secondary metabolic abnormalities | 0 | 0 |  |

Abbreviations: Phe, phenylalanine; Met, methionine; Cit, citrulline; Arg, Arginine; Tyr, Tyrosine; Pro, Proline; C0, free carnitine; C2, acetylcarnitine; C3, propionylcarnitine; C4, butyrylcarnitine; C5, isovalerylcarnitine/2-methylbutyrylcarnitine; C5OH, 3-hydroxy-isovalerylcarnitine; C6, Hexanoylcarnitine; C8, octanoylcarnitine; C10, decanoylcarnitine; C5DC, glutarylcarnitine/3-hydroxydecanoylcarnitine; C14, Tetradecanoylcarnitine; C16, palmityolcarnitine; C18, stearoylcarnitine; C10:2, Decadienoylcarnitine.

Note: Blood biochemical tests or enzyme activity tests, and urine organic acid analysis are conducted for clinical diagnosis based on different types of diseases.
