# Supplementary material for: Application of targeted high-throughput sequencing as a diagnostic tool for neonatal genetic metabolic diseases following tandem mass spectrometry screening
Source: Front Public Health. 2024 Dec 24;12:1461141. doi: 10.3389/fpubh.2024.1461141 (PMC11703805; doi:10.3389/fpubh.2024.1461141)
Supplement: Supplementary file 4 [file Table_4.DOC]

**Supplementary Table S4 Abnormal indicators and follow-up results in 1192 false-positive cases.**

| LC-MS/MS Main abnormal indicators | Abnormal range values (μmol /L) | Normal reference range (μmol /L) | Number of cases | Possible disorder (s) | Number of NGS positive | follow-up |
| --- | --- | --- | --- | --- | --- | --- |
| Phe | 137.28-327.59 | 20.00-120.00 | 176 | Phenylketonuria | Five cases of Phenylketonuria carriers | Normal Good growth and development, neurological function, and plasma Phe monitoring levels were normal. |
| Phe/Tyr | 3.6-7.07 | 0.20-1.20 |  |  |  |  |
| Met | 50.40-127.86 | 6.00-40.00 | 65 | Hyperhomocysteinemia; Methionine adenosyltransferase deficiency | One case of Citrin deficiency carrier | Growth and development as well as plasma levels of homocysteine and Met were within normal limits. Good growth and development, normal plasma Met levels. |
| Met/Phe | 0.79-2.24 | 0.12-0.70 |  |  |  |  |
| Cit | 36.34-244.36 | 6.50-35.00 | 114 | Citrin deficiency; argininosuccinate synthetase deficiency | One case of Citrin deficiency carrier | Growth Good growth and development, normal plasma liver function and Cit were within normal limits monitoring levels. |
| Cit/Phe | 0.69-5.16 | 0.12-0.65 |  |  |  |  |
| Cit | 2.78-5.8 | 6.50-35.00 | 24 | Ornithine transcarbamylase deficiency; | One case of Carbamogl phosphate synthe tose 1 carrier | Neurological development was normal and blood ammonia and urea levels were normal. |
|  |  |  |  | Carbamogl phosphate synthe tose 1 |  |  |
| Arg | 61-131.3 | 1.4-50 | 31 | Argininemia | 0 | Good growth and development, normal liver function and Arg levels. |
| Arg/Phe | 1.1-2.35 | 0-1 |  |  |  |  |
| Tyr | 354-603 | 24-307.9 | 18 | Tyrosinemia | 0 | Good growth and development, normal liver function, Tyr and urine acetone succinate levels. |
| Pro | 491-630 | 60-450 | 13 | Hyperprolinemia | 0 | Good growth and development with normal Pro levels. |
| C3 | 4.5-7.19 | 0.40-4.20 | 61 | Methylmalonic acidemia; Propionic acidemia | 0 | The nervous system developed normally, and blood C3 and urine levels of 3-hydroxypropionic acid and methylcitric acid were normal. |
| C3/C2 | 0.3-0.51 | 0.04-0.25 |  |  |  |  |
| C5DC | 0.56-2.15 | 0.04-0.25 | 51 | Glutaric acidemia type I | 0 | Normal neurological development, normal levels of C5DC in blood and glutaric acid in urine. |
| C5DC/C8 | 38.12-73.75 | 0.50-3 |  |  |  |  |
| C5OH | 0.89-3.68 | 0.08-0.45 | 98 | 3-Hydroxy-3-methylglutaryl CoA lyase deficiency; | Two cases of 3-Methylcrotonyl-CoA carboxylase deficiency carriers | Normal development of the nervous system and normal blood levels of C5OH. |
|  |  |  |  | 3-Methylcrotonyl-CoA carboxylase deficiency; |  |  |
| C5OH/C8 | 21.25-61.23 | 1.5-15 |  | Biotinidase deficiency |  |  |
| C0 | 5.21-9.62 | 10.00-60.00 | 239 | Primary carnitine deficiency | One case of Primary carnitine deficiency carrier | Normal blood levels of C0. |
| C4 | 0.67-2.79 | 0.07-0.45 | 36 | Isobutyryl-CoA dehydrogenase deficiency; | One case of Short-chain acyl-CoA dehydrogenase deficiency carrier | Normal growth and development, normal blood levels of C4. |
|  |  |  |  | Ethylmalonic encephalopathy; |  |  |
| C4/C3 | 0.27-2.29 | 0.03-0.16 |  | Short-chain acyl-CoA dehydrogenase deficiency |  |  |
| C8 | 0.21-1.25 | 0.01-0.17 | 32 | Medium chain acyl CoA dehydrogenase deficiency | 0 | Normal blood levels of C8. |
| C8/C2 | 0.03-0.33 | 0-0.02 |  |  |  |  |
| C8/C10 | 2.12-6 | 0.3-1.5 |  |  |  |  |
| C16 | 7.28-11.72 | 0.40-6.00 | 59 | Carnitine palmitoyltransferase II deficiency | 0 | Normal ability to perform daily activities, normal blood glucose, C14,16,18 levels. |
| C18 | 2.11-2.83 | 0.2-1.8 |  |  |  |  |
| C14 | 0.36-0.45 | 0.04-0.35 |  |  |  |  |
| C10:2 | 0.42-1.83 | 0.04-0.35 | 11 | 2,4-Dienoyl-CoA reductase deficiency | 0 | Good growth and development, normal blood levels of C10:2. |
| C5 | 0.31-2.56 | 0.03-0.26 | 117 | isovaleric acidemia | 0 | Good growth and development, with normal blood levels of C5 and urinary isovalerylglycine. |
| C5/C3 | 0.34-6.61 | 0.02-0.3 |  |  |  |  |
| Multiple amino acid or acylcarnitine abnormalities | | | 47 | Secondary metabolic abnormalities | 0 | Good growth and development, normal blood amino acids or acylcarnitines. |

Abbreviations: Phe, phenylalanine; Met, methionine; Cit, citrulline; Arg, Arginine; Tyr, Tyrosine; Pro, Proline; C0, free carnitine; C2, acetylcarnitine; C3, propionylcarnitine; C4, butyrylcarnitine; C5, isovalerylcarnitine/2-methylbutyrylcarnitine; C5OH, 3-hydroxy-isovalerylcarnitine; C6, Hexanoylcarnitine; C8, octanoylcarnitine; C10, decanoylcarnitine; C5DC, glutarylcarnitine/3-hydroxydecanoylcarnitine; C14, Tetradecanoylcarnitine; C16, palmityolcarnitine; C18, stearoylcarnitine; C10:2, Decadienoylcarnitine.
