# Supplementary material for: Application of targeted high-throughput sequencing as a diagnostic tool for neonatal genetic metabolic diseases following tandem mass spectrometry screening
Source: Front Public Health. 2024 Dec 24;12:1461141. doi: 10.3389/fpubh.2024.1461141 (PMC11703805; doi:10.3389/fpubh.2024.1461141)
Supplement: Supplementary file 5 [file Table_5.DOCX]

Supplementary Table S5 Analysis of gene variation in 98 cases of hereditary metabolic diseases.

| Types of diseases | Gene | | Variant alleles number | Nucleotide variant | Amino acid variant | Variation type | Pity analysis# | Reported | Cases |
| --- | --- | --- | --- | --- | --- | --- | --- | --- | --- |
| PCD |  | | 77 |  |  |  |  |  | 40 |
|  | *SLC22A5* | | 28 | c.51C>G | p. F17L | MV | P | Y | 22 |
|  |  | | 17 | c.1400C>G | p.S467C | MV | P | Y | 17 |
|  |  | | 10 | c.338G>A | p.C113Y | MV | P | Y | 8 |
|  |  | | 9 | c.428C>T | p.P143L | MV | P | Y | 8 |
|  |  | | 4 | c.760C>T | p.R254Ter | NV | P | Y | 4 |
|  |  | | 2 | c.1064C>T | p.S355L | MV | VUS | Y | 2 |
|  |  | | 3 | c.1195C>T | p.R399W | MV | P | Y | 3 |
|  |  | | 1 | c.904A>G | p.K302E | MV | VUS | Y | 1 |
|  |  | | 1 | c.1412G>A | p.R471H | MV | P | Y | 1 |
|  |  | | 1 | c.797C>T | p.P266L | MV | P | Y | 1 |
|  |  | | 1 | c.415G>A | p.D139N | MV | VUS | Y | 1 |
| SCAD |  | | 10 |  |  |  |  |  | 5 |
|  | *ACADS* | | 3 | c.1031A>G | p.E344G | MV | P | Y | 2 |
|  |  | | 1 | c.293A>G | p.Y98C | MV | VUS | Y | 1 |
|  |  | | 1 | c.319C>T | p.R107C | MV | P | Y | 1 |
|  |  | | 1 | c.628G>A | p.E228K | MV | VUS | N | 1 |
|  |  | | 1 | c.79A>C | p.T27P | MV | VUS | N | 1 |
|  |  | | 1 | c.981_983del | p.T328del | IV | VUS | Y | 1 |
|  |  | | 1 | c.625G>A | p.G209S | MV | B | Y | 1 |
|  |  | | 1 | c.988C>T | p.R330C | MV | P | Y | 1 |
| CPT-II |  | | 4 |  |  |  |  |  | 2 |
|  | *CPT2* | | 3 | c.1102G>A | p.V369I | MV | B | Y | 2 |
|  |  | | 1 | c.1055T>G | p.F352C | MV | LB | Y | 1 |
| MCAD | *ACADM* | | 1 | c.424_426del | p.Lys144del | IV | LP | Y | 1 |
|  |  | | 1 | c.668T>C | p.I223T | MV | P | Y | 1 |
| PAHD |  | | 34 |  |  |  |  |  | 17 |
|  | *PAH* | | 9 | c.728G>A | p.R243Q | MV | P | Y | 7 |
|  |  | | 5 | c.611A>G | p.Y204C | MV | P | Y | 4 |
|  |  | | 4 | c.158G>A | p.R53H | MV | VUS | Y | 4 |
|  |  | | 2 | c.754C>T | p.R252W | MV | P | Y | 1 |
|  |  | | 1 | c.547G>T | p.E183Ter | NV | P | N | 1 |
|  |  | | 1 | c.442-1G>A | p.?/IVS4-1G>A | SV | P | Y | 1 |
|  |  | | 1 | c.782G>A | p.R261E | MV | P | Y | 1 |
|  |  | | 1 | c.842+1G>A | p.?/IVS7+1G>A | SV | P | Y | 1 |
|  |  | | 1 | c.1238G>C | p.R413P | MV | P | Y | 1 |
|  |  | | 1 | c.251A>G | p.D84G | MV | P | Y | 1 |
|  |  | | 1 | c.875C>T | p.P292L | MV | LP | Y | 1 |
|  |  | | 1 | c.948delA | p.E316Ter | FV | P | N | 1 |
|  |  | | 1 | c.1174T>A | p.F392I | MV | P | Y | 1 |
|  |  | | 1 | c.168+1G>A |  | SV | P | Y | 1 |
|  |  | | 1 | c.707-1G>A | IVS6-1G>A | SV | P | Y | 1 |
|  |  | | 1 | c.722delG | p.R241fs | FV | P | Y | 1 |
|  |  | | 1 | c.617A>G | p.Y206C | MV | P | Y | 1 |
|  |  | | 1 | c.907delT | p.S303PfsTer38 | FV | P | Y | 1 |
| BH4D |  | | 9 |  |  |  |  |  | 5 |
|  | *PTS* | | 5 | c.259C>T | p.P87S | MV | P | Y | 3 |
|  |  | | 2 | c.155A>G | p.N52S | MV | P | Y | 1 |
|  |  | | 1 | c.331G>A | p.A111T | MV | P | Y | 1 |
|  |  | | 1 | c.286G>A | p.D96N | MV | P | Y | 1 |
| CD |  | | 15 |  |  |  |  |  | 9 |
|  | *SLC25A13* | | 10 | c.852_855del | p.M285PfsTer2 | FV | P | Y | 6 |
|  |  | | 2 | c.851_854del | p.M285Pfs | FV | P | Y | 1 |
|  |  | | 1 | c.1364G>C | p.R455P | MV | VUS | N | 1 |
|  |  | | 1 | c.1067G>A | p.R356Q | MV | P | Y | 1 |
|  |  | | 1 | c.615+5G>A |  | SV | P | Y | 1 |
| CPS I |  | | 6 |  |  |  |  |  | 3 |
|  | *CPS1* | | 4 | c.1030A>G | p.T344A | MV | B | Y | 2 |
|  |  | | 1 | c.952C>A | p.Q318K | MV | P | Y | 1 |
|  |  | | 1 | c.950G>A | p.G317E | MV | VUS | Y | 1 |
| MAT I/III | |  | 4 |  |  |  |  |  | 2 |
|  | | *MAT1A* | 2 | c.755T>C | p.I252T | MV | VUS | Y | 2 |
|  | |  | 1 | c.406-8C>T |  | SV | LB | Y | 1 |
|  | |  | 1 | c.754A>T | p.I252F | MV | VUS | Y | 1 |
| 3MCC | |  | 9 |  |  |  |  |  | 5 |
|  | | *MCCC1* | 3 | c.639+2T>A | IVS6dsT-A+2 | SV | P | Y | 2 |
|  | |  | 1 | c.196C>T | p.R66C | MV | LP | Y | 1 |
|  | |  | 1 | c.1582C>A | p.L528I | MV | VUS | Y | 1 |
|  |  | | 1 | c.863A>G | p.E288G | MV | LP | Y | 1 |
|  |  | | 1 | c.493A>C | p.T165P | MV | LP | N | 1 |
|  | *MCCC2* | | 1 | c.538C>T | p.R180Ter | NV | P | Y | 1 |
|  |  | | 1 | c.351_353del | p.G118del | IV | LP | Y | 1 |
| GA-Ι |  | | 6 |  |  |  |  |  | 3 |
|  | *GCDH* | | 2 | c.1244-2A>G |  | SV | P | Y | 1 |
|  |  | | 1 | c.700C>T | p.R234W | MV | P | Y | 1 |
|  |  | | 1 | c.1063C>T | p.R355C | MV | P | Y | 1 |
|  |  | | 1 | c.1045G>A | p.A349T | MV | P | Y | 1 |
|  |  | | 1 | c.1060G>A | p.G354S | MV | P | Y | 1 |
| MMA |  | | 6 |  |  |  |  |  | 3 |
|  | *MMAA* | | 2 | c.742C>T | p.Q248Ter | NV | P | Y | 1 |
|  | *MMACHC* | | 1 | c.398_399del | p.Q133RfsTer5 | FV | P | Y | 1 |
|  |  | | 1 | c.609G>A | p.W203X | NV | P | Y | 1 |
|  | *HCFC1* | | 2 | c.5189G>A | p.S1730A | MV | VUS | Y | 1 |
| IVA | *IVD* | | 1 | c.148C>T | p.Arg50Cys | MV | LP | Y | 1 |
|  |  | | 1 | c.1199A>G | p.Tyr400Cys | MV | P | Y | 1 |
| BTDD | *BTD* | | 2 | c.1211G>C | p.C404S | MV | P | Y | 1 |
| PA | *PCCB* | | 2 | c.1087T>C | p.S363P | MV | LP | Y | 1 |

Abbreviations: LP, Likely pathogenic; P, Pathogenic; VUS, uncertain significance; B, Benign; LB, Likely Benign; N, no; Y, yes; MV, missense variant; NV, nonsense variant; IV, inframe variant; FV, frameshift variant; SV, splicing variant; CPT-II, carnitine palmitoyltransferase II deficiency; CPS-I, Carnitine palmitoyltransferase I deficiency; MAT I/III, Methionine adenosyltransferase deficiency; PAHD, Phenylalanine hydroxylase deficiency; BH4D, Tetrahydrobiopterin deficiency; CD, Citrin deficiency; MMA, Methylmalonic acidemia; PA, Propionic acidemia; IVA, isovaleric acidemia; GA-Ι, Glutaric acidemia type I; 3MCC, 3-Methylcrotonyl-CoA carboxylase deficiency; BTDD, Biotinidase deficiency; PCD, Primary carnitine deficiency; SCAD, Short-chain acyl-CoA dehydrogenase deficiency; MCAD, Medium chain acyl CoA dehydrogenase deficiency; Genome reference hg19/GRCh37.
